# Difference in Cerebral Microembolization With Multielectrode Pentaspline or Variable‐Loop Circular PFA Systems

**DOI:** 10.1111/jce.70218

**Published:** 2025-12-08

**Authors:** Mattia Pagnoni, Leonardo Caranzano, Cheryl Teres, Ciro Ascione, Mathieu Le Bloa, Panagiotis Antiochos, Adrian Luca, Jorge Solana‐Muñoz, Diana Ortolani, Daniel Andrade Azevedo, Etienne Pruvot, Lorenz Hirt, Patrizio Pascale

**Affiliations:** ^1^ Division of Cardiology Lausanne University Hospital and University of Lausanne Lausanne Switzerland; ^2^ Division of Neurology Lausanne University Hospital and University of Lausanne Lausanne Switzerland

**Keywords:** AF ablation, atrial fibrillation, cerebral microembolism, PFA, pulmonary vein isolation, stroke

## Abstract

**Background:**

Pulsed field ablation (PFA) is a largely nonthermal modality with myocardial selectivity and collateral damage sparing. Cerebrovascular safety remains an important consideration as catheter design and pulse parameters may influence embolic risk. Microembolic signals (MES), detected by transcranial Doppler (TCD) ultrasonography, may offer a sensitive real‐time surrogate for procedural‐related embolic activity.

**Methods:**

We prospectively compared MES burden between two PFA systems: a multielectrode Pentaspline catheter (Farawave, Boston Scientific) and a more recently introduced variable‐loop circular catheter (VLCC) (Varipulse, Biosense Webster). Consecutive patients undergoing first‐time pulmonary vein isolation for AF were included. ACT targets were > 300 s (Pentaspline) and > 350 s (VLCC), and ablation was delivered according to manufacturer‐recommended workflows.

**Results:**

Among 23 patients (16 Pentaspline, 7 VLCC), ACT was maintained above respective targets in all, and left atrial dwell time was comparable. The total number of PFA deliveries was significantly higher in the Pentaspline group compared to the VLCC group (51 [46–66] vs. 22 [22–25], *p* < 0.001). The MES burden was significantly higher with VLCC during ablation time (919 [154–2302] vs. 102 [52–248], *p* = 0.005). This difference was even higher when normalized by the delivered‐to‐recommended PFA deliveries ratio (*p* = 0.005). In addition, MES burden with VLCC displayed markedly greater inter‐patient variability, whereas Pentaspline results were more reproducible (IQR 2148 vs. 196, *p* < 0.001).

**Conclusions:**

VLCC PFA was associated with higher and more variable cerebral microembolization compared with the Pentaspline system. These findings underscore the impact of catheter design and energy delivery on embolic load and highlight the need for dedicated cerebrovascular safety evaluation.

Pulsed field ablation (PFA) is a novel, largely nonthermal ablation modality with unique biophysics such as cardiac tissue selectivity and collateral damage sparing. A sizeable body of clinical evidence supports the safety and efficacy of PFA for pulmonary vein isolation (PVI), including the lack of esophageal injury. Cerebrovascular safety remains an important consideration as catheter design, electrode configuration, and pulse delivery parameters may influence embolic risk, particularly in the form of silent cerebral lesions (SCL) [1, 2]. In that respect, a more sensitive metric of the embolic risk may be desirable to anticipate even subtle differences in the risk of cerebral embolism. Microembolic signals (MES), detected by transcranial Doppler (TCD) ultrasonography, may offer a sensitive real‐time surrogate for procedural‐related embolic activity.

We prospectively compared MES burden between two PFA systems: a multielectrode Pentaspline catheter (Farawave, Farapulse‐Boston Scientific) and a more recently introduced variable‐loop circular catheter (VLCC) (Varipulse, Biosense Webster). Consecutive patients undergoing first‐time PVI for AF were included. All patients were treated with a direct oral anticoagulant. All procedures were performed under transesophageal echocardiography with minimally interrupted anticoagulation. ACT targets were > 300 s (Pentaspline) and > 350 s (VLCC). Ablation was delivered according to manufacturer‐recommended workflows: 8‐application‐per‐PV protocol in the Pentaspline group with each delivery consisting of five bipolar pulses, and four deliveries per vein in the VLCC group with each delivery consisting of three bipolar pulses separated by ≥ 10‐s delays. In case of electrode overlap, distal and proximal electrodes were deactivated for the VLCC catheter, in accordance with manufacturer recommendations.

Continuous TCD monitoring of the middle cerebral artery was initiated after transseptal access and maintained until catheter withdrawal using a 1.5‐MHz probe (TCD‐X Doppler system, Atys Medical, France). All Doppler signals underwent automated processing by the system's proprietary algorithm, and all signals not classified as artifact by the system were manually reassessed offline by a blinded experienced operator according to international consensus recommendations [3]. Differentiation between solid and gaseous signals was not performed, as the 1.5‐MHz probe and the signal‐processing capabilities of the system do not allow reliable discrimination. All qualifying signals were therefore counted as MES. To estimate the embolic activity per unit of energy delivery and account for differences between the two systems in the number of applications recommended to achieve PVI, the total number of MES recorded during ablation was divided for each patient by (1) the number of PFA pulses, (2) the number of PFA deliveries, and (3) the manufacturer‐recommended number of (minimal) PFA deliveries to achieve PVI (the “delivered‐to‐recommended PFA deliveries ratio”). The study was approved by the institutional ethical review board, and patients provided written informed consent.

Twenty‐three consecutive patients were monitored (Pentaspline *n* = 16, VLCC *n* = 7). Median age was 59 [56–67] years, and 70% were male (Table 1). Baseline characteristics were comparable, apart from older age in the Pentaspline group (63 [56–69] vs. 56 [55–59], *p* = 0.034). ACT was maintained above respective targets in all.

**Table 1:** Clinical, procedural, and microembolic characteristics in the overall population and between‐group comparisons of Pentaspline versus VLCC catheters.

	Total (*n* = 23)	Pentaspline (*N* = 16)	VLCC (*N* = 7)	*p* value
Patient characteristics				
Age, mean (± SD), years	59 [56–67]	63 [56–69]	56 [55–59]	0.034
Gender, male, *n* (%)	16 (70%)	12 (75%)	4 (57%)	0.392
Paroxysmal AF, *n* (%)	15 (65%)	9 (56%)	6 (86%)	0.172
CHADS‐VASC	1 [0–3]	1 [0–3]	1 [0–2]	0.703
BMI, median (IQR), kg/m^2^	28 [23–35]	29 [24–37]	24 [22–28]	0.071
LVEF, median (IQR), %	63 [59–67]	61 [55–65]	66 [61–68]	0.096
LA volume, median (IQR), mL	60 [46–80]	62 [54–93]	54 [35–64]	0.096
LA volume index, median (IQR), mL/m^2^	32 [25–42]	38 [26–46]	28 [21–31]	0.074
Previous stroke, *n* (%)	4 (17.4%)	3 (18.8%)	1 (14.3%)	0.795
Known carotid or aortic atheromatous disease	1 (4.3%)	0 (0%)	1 (14.3%)	0.122
Procedural characteristics				
Intraprocedural ACT, median (IQR), s	463 [394–509]	448 [374–508]	494 [444–522]	0.29
Number of PFA deliveries, median (IQR), *n*	46 [25–58]	51 [46–66]	22 [22–25]	< 0.001
Number of PFA pulses, median (IQR), *n*	230 [75–290]	255 [230–330]	66 [66–75]	< 0.001
Posterior wall ablation, *n* (%)	2 (8.7%)	1 (6.3%)	1 14.3%)	0.529
Ratio delivered‐to‐recommended PFA deliveries, median (IQR)	1.5 [1.4–1.8]	1.6 [1.4–2.1]	1.4 [1.4–1.6]	0.055
Total ablation time, median (IQR), min	27.8 [23.1–44.8]	25.7 [21.9–42.8]	32.9 [27.9–49.9]	0.038
Total left atrial dwelling time, median (IQR), min	52.1 [41.4–60.9]	49.8 [35.8–62.7]	54.6 [49.5–60.9]	0.385
MES findings				
MES during total left atrial dwelling time, median (IQR), *n*	169 [120–349]	157 [108–279]	1027 [167–2453]	0.013
MES during ablation, median (IQR), *n*	154 [91–313]	102 [52–248]	919 [154–2302]	0.005
Ratio MES during ablation/number of PFA deliveries, median (IQR)	4.3 [1.4–13.7]	2.1 [1.1–4.7]	41.8 [7–104.6]	< 0.001
Ratio MES during ablation/number of PFA pulses, median (IQR), *n*	0.9 [0.3–2.7]	0.4 [0.2–1]	13.9 [2.3–34.9]	< 0.001
Ratio MES during ablation/delivered‐to‐recommended PFA deliveries ratio, median (IQR)	112 [43.3–250.4]	68.2 [33.6–151.9]	668.4 [112–1674.2]	0.005

Abbreviations: ACT = activated clotting time, AF = atrial fibrillation, LA = left atrium, LVEF = left ventricle ejection fraction, MES = microembolic signals, VLCC = variable‐loop circular catheter.

The total number of PFA deliveries and pulses was significantly higher in the Pentaspline group compared to the VLCC group (51 [46–66] vs. 22 [22–25] and 255 [230–30] vs. 66 [66–75], *p* < 0.001 for both). Additional posterior wall ablation was performed in one patient in each group (*p* = 0.529). The mean delivered‐to‐recommended PFA deliveries ratio showed a trend towards higher values in the Pentaspline group compared to the VLCC group (1.6 [1.4–2.1] vs. 1.4 [1.4–1.6], *p* = 0.055). The total left atrial dwell time was comparable between the two groups (49.8 [35.8–62.7] vs. 54.6 [49.5–60.9] min, *p* = 0.385).

Continuous TCD monitoring showed a significantly higher MES burden during total left atrial dwell time in the VLCC group compared to the Pentaspline group (1027 [167–2453] vs. 157 [108–279]; *p* = 0.013). The difference was related to markedly higher MES counts during ablation time with VLCC (919 [154–2302] vs. 102 [52–248], *p* = 0.005) (Figure 1A). This difference was even higher when normalized by the number of PFA deliveries and pulses (*p* < 0.001), or the delivered‐to‐recommended PFA deliveries ratio (*p* = 0.005). In addition to higher counts, MES burden with VLCC displayed markedly greater inter‐patient variability, whereas Pentaspline results were more reproducible (IQR 2148 vs. 196; SD 1078 vs. 187, *p* < 0.001) (Figure 1B).

**Figure 1:**
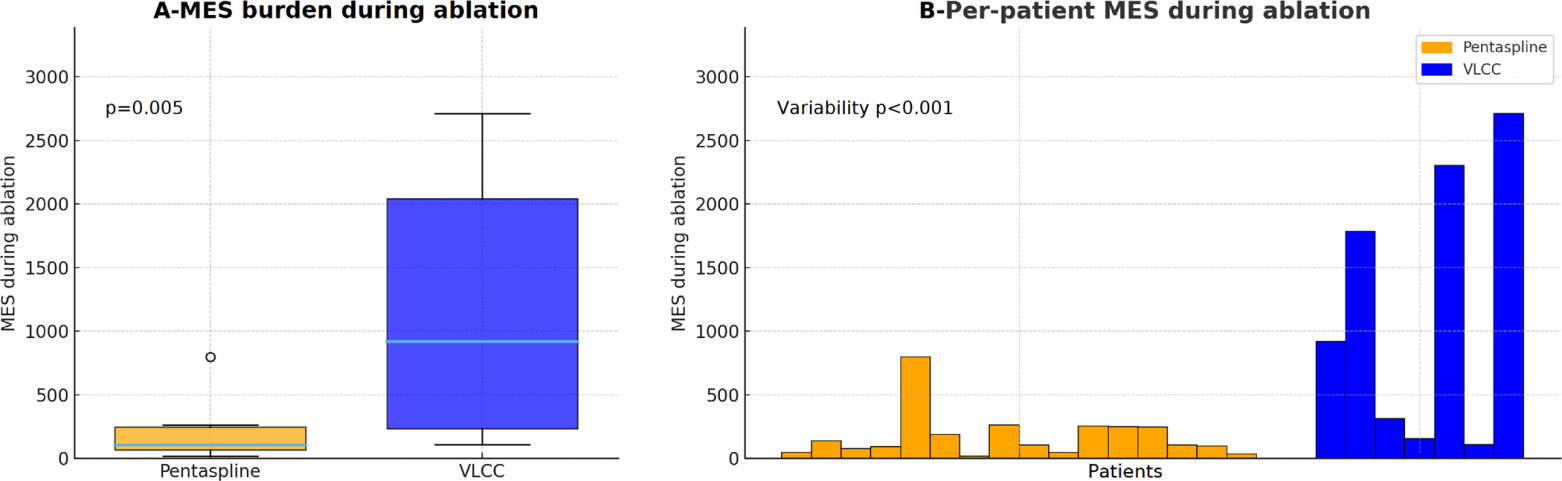
(A) Comparative cerebral microembolic burden during ablation with Pentaspline versus VLCC catheters. (B) Individual per‐patient MES burden observed during ablation.

MES burden differed markedly between the two PFA systems, with approximately tenfold higher burden with the VLCC as compared to the Pentaspline group when normalized to the number of delivered‐to‐recommended PFA deliveries. This finding was not explained by differences in baseline characteristics, as patients in the VLCC group were younger with a trend towards smaller left atria and more frequent paroxysmal forms of AF. Another important finding of the study is the marked inter‐patient heterogeneity in MES counts among patients in the VLCC group. Such variability suggests that embolic load may involve a less predictable mechanism, which may be less easily reproduced in ex vivo models, such as stacked sequential applications, electrode overlap, or positioning in low blood flow regions with reduced passive cooling.

A recent study by Miyazaki et al. based on post‐procedural brain MRI is consistent with our MES‐based observations. They reported a higher incidence and size of SCL following PFA using the VLCC compared to an over‐the‐wire type multielectrode array catheter (PulseSelect; Medtronic) [2]. Sauer et al. recently reported the observation of consistent electrode heating and heat stacking with VLCC PFA applications in an in vitro model. The amount of heating was significantly reduced by increasing irrigation to 30 mL/min, instead of the 4 mL/min irrigation rate used in previous clinical studies [1, 2, 4]. The role of active electrode irrigation during PFA to improve safety and reduce the risk of embolic events must, however, be evaluated.

This analysis has important limitations. First, the limited sample size and single‐center non‐randomized design restrict generalizability. Second, MES detected by TCD represent a surrogate of cerebral embolic activity and cannot be equated with SCL or clinically relevant cerebrovascular events. However, modifying workflows or identifying causes of increased MES is, anyhow, a desirable goal since all MES are potential embolic sources. This was illustrated with the initial use of multielectrode phased radiofrequency catheters. Studies showed that by deactivating certain electrodes, the number of Doppler‐detected embolic signals decreased, which translated in a reduction of the number of MRI‐detected SCL [5, 6]. Finally, procedures were performed using the irrigation settings available at the time (4 mL/min for VLCC); the present findings may therefore not fully reflect current workflows.

This preliminary analysis demonstrates higher and more variable MES burden with the VLCC compared to the Pentaspline system. Although the clinical significance of MES remains uncertain, these findings underscore the influence of catheter design and pulse delivery parameters on embolic load. A cautious approach is therefore warranted, and further studies incorporating integrated neuroimaging are needed to better define the cerebrovascular safety profile of each PFA system.

## Conflicts of Interest

There was no industry support for this study. Dr Pascale has received research grants from the Swiss National Science Foundation and the Michel Tossizza Foundation, all for work outside of the submitted study, and has received speaker honoraria from Boston Scientific and travel grants from Biosense Webster and Boston Scientific. Dr Teres has received travel grants, speaker honoraria, and consultant fees from Biosense Webster and Boston Scientific. Dr Pagnoni received travel grants and educational grants from Biosense Webster. The other authors have nothing to disclose.
